# Interrelationships among mountain relief, surface organic layer, soil organic carbon, and its mineral association under subarctic forest tundra

**DOI:** 10.1038/s41598-022-21521-9

**Published:** 2022-10-14

**Authors:** Viliam Pichler, Erika Gömöryová, Ján Merganič, Peter Fleischer, Marián Homolák, Alexander Onuchin, Jozef Výbošťok, Konstantin Prosekin

**Affiliations:** 1 Faculty of Forestry, Technical University in Zvolen, T. G. Masaryka 24, 96001 Zvolen, Slovak Republic; 2 V.N. Sukachev Institute of Forest SB RAS, Akademgorodok 50, Building 28, 660036 Krasnoyarsk, Russian Federation; 3 Taimyr Directorate of Nature Reserves, Kirov Str. 24, 663305 Norilsk, Russian Federation

**Keywords:** Biogeochemistry, Carbon cycle

## Abstract

Efforts to estimate the impact of climate change-induced forest expansion on soil carbon stocks in cold regions are hindered by the lack of soil organic carbon (SOC) concentration data. The presented study addressed the information gap by establishing SOC concentration and its variability in two catchments inside the vast, remote, and rugged Putorana Plateau. Additionally, it explored interrelationships among the terrain relief, vegetation cover, surface organic layer, SOC and its mineral association on the northernmost boundary of the forest-tundra biome traversing the northwestern part of the Central Siberian Tableland. Soil samples were taken from the active layer on the slope base, middle, and below the upper forest boundary. Subsequently, they were analyzed for SOC concentration by dry combustion. Multiple linear regression identified associations between slope angle and surface organic layer thickness and between SOC concentration and surface organic layer thickness, clay content, and dithionite-extracted Al. Clay content and surface organic layer thickness explained 68% of the overall SOC concentration variability. When used with data produced by remote sensing-based multipurpose large-scale mapping of selected biophysical factors, the acquired regression equations could aid the estimation of SOC across the rugged terrain of the Siberian Traps.

## Introduction

Current warming in the Arctic and subarctic regions exceeds the global average and affects rock weathering, soil processes, vegetation dynamics, and atmospheric and soil carbon sequestration^1–4^. The persistent lack of soil organic carbon (SOC) data from high-latitude regions limits the confidence in predictions of the potential impacts of climate change on SOC and needs to be addressed^5–7^. Marked disparities in SOC stock estimates for boreal territories result from uncertainties in SOC concentration rather than bulk density^8^. In addition, SOC and its association with determining site conditions and mineral soil fractions of the Arctic and subarctic regions have mainly been studied in comparatively flat tundra terrains, and there is an information gap regarding their relationship in subarctic mountains featuring extensive slope positions. For example, there is a strong relationship between slope angle, solar irradiance at high latitudes^9^, and soil temperature^10^. Furthermore, even the mild topography of the Siberian tundra has been shown to affect the surface organic layer (SOL) thickness, its thermal insulation properties, the active layer depth, and carbon cycling^11^. Thermal insulation provided by the SOL reduced the mean annual temperature of the underlying mineral soil by 3 °C^12^. Lower temperatures diminish the weathering rate of basalt and soil and thus also affect the availability of reactive Fe and Al pedogenic minerals that enable the retention and stabilization of SOC^13–15^. The effect of temperature on pedogenesis is partly indirect, as temperature affects the amount of water available for soil-forming processes, including soil weathering and leaching in cold regions^16–20^. A thick soil organic layer with sizeable water storage may lead to decreased availability of liquid water in the mineral soil and thus a lower weathering rate. For example, spruce tree litter may store up to 4.4 mm H_2_O in the 1 cm layer^21^.

Therefore, the presented research focused on the SOC concentration and its association with important biophysical factors and soil weathering products on extended, variably steep slopes of the vast and remote Putorana Plateau, one of the least investigated subarctic areas. The basaltic plateau encompasses approximately 250,000 km^2^ in the NW part of the Central Siberian Tableland^22^. Spatial correlation between SOC and Fe and Al oxide and hydroxide concentrations identified in an adjacent section of the Yenisei River basin^23^ showed that secondary Fe and Al minerals could promote SOC stabilization in the adjacent Putorana massif itself. Their abundance in Putorana soils resulted from basalt weathering and the replacement of the original basaltic glass by amorphous material rich in Si^4+^, Al^3+^ and Fe^3+^^24–26^. However, only a portion of SOC is associated with the soil mineral fraction and particulate organic matter, formed by tiny fragments of plants, and microbial residues^27^ also contribute to the bulk SOC content. In Arctic permafrost soils, approximately 54% of the total SOC is bound to the mineral pool^28^.

Given the present lack of information on SOC concentration patterns in subarctic mountainous landscapes, the main study objective was to reveal potential SOC concentration trends according to differential factors gaining prominence in rugged terrains, mainly the slope angle, vegetation, and SOL. Given the relatively homogenous lithology of the Putorana Plateau, we formulated two working hypotheses: (i) SOC concentration is affected by one or several biophysical factors specific to subarctic mountain terrains; and (ii) SOC concentration is strongly associated with pedogenic Fe and Al minerals. Since our study was part of a broader investigation focusing on forest-tundra ecosystems that include spruce (*Picea* sp.), it was limited to southerly slopes with a comparatively higher share of Siberian spruce (*Picea obovata* Ledeb.)

## Materials and methods

Our research was conducted approximately 250–320 km above the Arctic circle, approximately 150 km deep inside the Putorana Plateau, which is the most elevated NW part of the Central Siberian Tableland^29^.

### Selection and description of research localities

**Figure 1:**
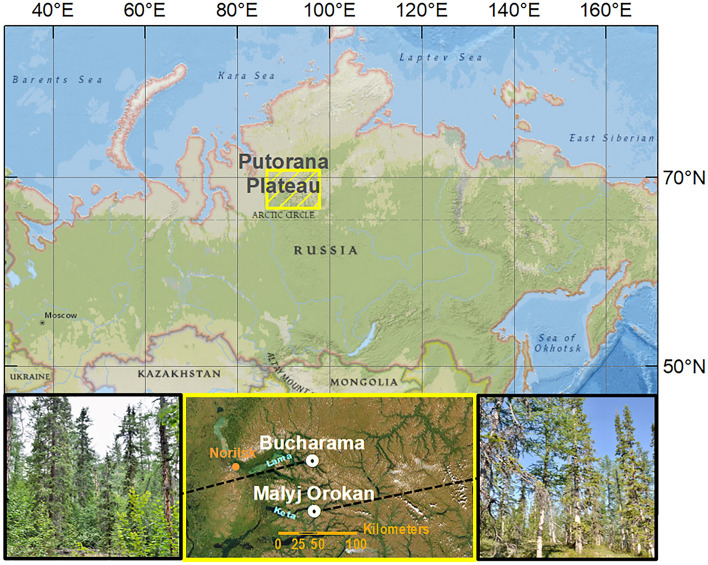
Position of the Bucharama and Malyj Orokan valleys intersecting the Putorana Plateau. The map was created using ArcGIS 10.2 © 1995–2022 ESRI Inc., licensed under the Esri Master License Agreement (https://www.esri.com/en-us/legal/terms/full-master-agreement).

The Putorana Plateau is the largest monolithic mountain range and World Heritage Site in the Russian polar region, extending from 89° to 101°E and from 67° to 71°N (Fig. 1). Multiple uplifts on the basaltic Putorana Plateau have generated flat-topped landscapes averaging 900–1200 m in elevation and deep valleys, featuring the trappean structure of slopes with several levels^6,30,31^. Siberian basalts are very homogeneous in chemical and mineralogical compositions and are represented by tholeiites, typical for all trap formations of the world, with high and spatially consistent contents of iron and aluminum oxides^32^.

Two research sites with distinct average slope angles were selected on southerly slopes overlooking deep valleys of the Bucharama (69°28′2.86″N, 91°25′56.16″E) and Malyj Orokan (68°44′48.18″N, 91°32′8.34″E) Rivers at altitudes of 130 m–430 m a.s.l. and 100 m–360 m a.s.l., respectively. The respective mean annual temperatures and precipitation amounts for the two localities were similar, i.e., − 9.4 °C and 435.3 mm for Bucharama and − 10.1 °C and 456.7 mm for Malyj Orokan. The average values were calculated based on the period 1901–2020 from Version 4 of the CRU TS Monthly High-Resolution Gridded Multivariate Climate Dataset^33^. Importantly, the differences in slope angle and its standard deviation (SD) between the former (avg. = 28.8°, SD = 15.6°) and the latter locality (avg. = 10.6°, SD = 6.7°) were significant (p < 0.01). The cold subarctic climate sustains a continuous permafrost table at a 1–3 m depth and widespread development of cryogenic processes, e.g., solifluction^29^. The geological bedrock was formed by flow basalts at both localities. Soil cover comprised basalt-derived Eutric Cambisols (according to World Reference Base^34^) with 40–50% stony content. They formed after the last deglaciation 25–10 thousand years ago^29^ in drained positions on the slopes under spruce-larch forest-tundra, with mossy-lichen and mossy-herbaceous ground cover and without eluvial-illuvial redistribution of clay. Previous results showed that intense weathering of basalt-derived plagioclases and vitreous minerals was accompanied by the leaching of silica and calcium, coagulation-cryochemogenic granulation, and the accumulation of SOC, Fe, and Al^35–38^. Vegetation cover in both localities was dominated by spruce (*Picea obovata* Ledeb.) and larch (*Larix sibirica* Ledeb., *Larix gmelinii* Rupr.) and interspersed by birch (*Betula* sp.). The undergrowth and ground cover contained blueberry, Siberian currant, mosses, and lichens. The forest-tundra upper boundary reached approx. 500 m a.s.l. The selection of the two localities for soil sampling ensured that most site conditions were relatively constant regarding climate and lithology.

### Soil sampling and analyses

In each of the two localities, three horizontal transects, each approximately 250–300 m long, were established on predominantly southerly slopes. The transects were placed along contour lines on the foot slopes, in the middle of the forested slope parts, and below the upper forest boundary at the following elevations: 130 m, 250 m, and 430 m a.s.l. in the Bucharama valley and 100 m, 260 m, and 360 m a.s.l. in the valley of Malyj Orokan. Each transect contained five soil pits and pedological profiles, approx. 50–60 m apart. They were excavated and exposed to a depth of 30–40 cm from the mineral soil surface. The average thickness of the SOL composed of dry lichen, mosses, and forest litter was recorded on the soil profile wall by a ruler. Soil samples, each weighing approx. 150 g, were taken from 10 cm, 20 cm, and 30 cm depths. During the summer 2018 and 2019 sampling campaigns, soil samples were stored in base camps in partly open plastic bags. After that, they were airlifted to laboratories for further physical and chemical analyses.

Soil texture was determined by the pipette method after the removal of organic compounds with hydrogen peroxide (H_2_O_2_) and clay dispersion^39^. Soil pH was measured in a deionized water suspension of air-dried soil at a soil-to-solution ratio of 1:2.5 by a pH-meter instrument. The contents of C and N in the fine earth (< 2 mm) were obtained through the dry combustion method using a CN analyzer (Vario Isotope Cube, Elementar Analysis Systems GmbH, Hanau, Germany). The C/N ratio was calculated as an indicator of particulate organic matter presence (> 10), as opposed to minerally associated organic matter (< 13)^40^. Free Fe (Fe o, Fe d) and Al (Al o, Al d) contained in the fine earth fraction were obtained by parallel extractions using 0.2 M ammonium oxalate and sodium dithionite–citrate solutes, respectively^41^. Dithionite-citrate extraction represents both crystalline and poorly crystalline Fe oxides (Fe d) and the amount of Al substituted in Fe oxides and hydroxides (Al d)^42–44^. Al substitution in iron oxides indicates more favorable weathering conditions^45^. Oxalate-extractable Fe and Al represent poorly crystalline aluminosilicates and ferrihydrite, as well as Al and Fe in organic complexes^42,43^. Barium chloride (BaCl_2_), 0.1 M, was used to extract exchangeable Ca, Mg, and K^46^. Free Fe, Al, and exchangeable cation concentrations were measured by an inductively coupled plasma-optical emission spectrometer (ICP–OES Agilent 5100, USA).

### Aboveground forest biomass

The height, diameter, and crown diameter of trees were determined within 15-m-radius circles projected around each soil profile. Subsequently, we used previously published models for the total aboveground biomass of living trees, including stems, branches, twigs, and foliage, from similar geographical regions^47–50^. The aboveground biomass of trees with > 7 cm diameter at 1.3 m height was calculated using the diameter as the sole predictor for spruce or using both the diameter and the height for larch, birch, alder, willow, and rowan. For trees < 7 cm in diameter, the aboveground biomass was established as a function of tree height and crown diameter.

### Statistical analyses

A two-tailed Lilliefors test was used to test the null hypothesis that samples originated from a normally distributed population with an unknown mean and standard deviation. Differences between site conditions and soil properties (Table 1) at the two localities (Bucharama, Malyj Orokan) were assessed by a two-tailed t test or Mann‒Whitney test for two independent samples. Calculations were performed in IBM SPSS Statistics v. 28.0.1.0. In these and further analyses, the depth-averaged values for SOC concentration, clay content, and all other soil properties were calculated based on the data from all three surveyed depths (10 cm, 20 cm, and 30 cm). In other words, there were 15 average values representing each of the two localities. Separate-slopes ANCOVA was used to determine whether the locality as an independent categorical variable adjusted for covariates, i.e., altitude, slope angle, aboveground biomass, and SOL thickness, had a significant effect on the cube root-transformed SOC concentration data. The respective transformation was applied to ensure the homogeneity of variance, evaluated by Levene's test. Subsequently, multiple linear regression (MLR) with backward stepwise selection^51^ was applied to analyze relationships between dependent and explanatory variables. The purpose of the analysis was to identify a set of independent variables making a statistically significant contribution to the amount of SOC concentration variation explained by the model. The variance inflation factor (VIF) was determined for variables entering MLR. In the case of VIF > 5, ridge regression was used to account for multicollinearity^52^. In addition, the feasibility of the final MLR model was reviewed based on the partial correlations between each independent variable and the dependent variable after controlling for all other independent variables entering MLR. Each variable entering ANCOVA or MLR contained pooled data from both localities. All MLRs and ANCOVAs were performed in Statistica 12 (StatSoft, Inc., Tulsa, Oklahoma). Regression equations for the pairwise relationships between dependent (SOL thickness, SOC concentration) and explanatory variables (slope angle, clay content, Al o, Al d) were obtained by a curve fitting routine (LinearModelFit, NonlinearModelFit) available in Wolfram Mathematica 12.2.0 (Wolfram Research, Inc., Champaign, Illinois). The Akaike information criterion (AIC)^53^ and root mean squared error (RMSE) were reported for both linear and nonlinear (exponential, logarithmic, or sigmoid) models to assess possible nonlinearity in variables of the analyzed sample relationships. A threshold AIC difference Δ_AIC_ ≥ 10 between AIC values of linear and nonlinear models^54^ was used to select models featuring a lower AIC as more adequate than the others. The results were considered statistically significant if the level of significance was p < 0.05.

**Table 1:** Physical and chemical properties of soils in Bucharama (BU) and Malyj Orokan (MO).

Depth (cm)	Site	CL	Sand	Silt	Clay	pH H_2_O	SOCC (%)	C/N	HW (%)	Ca^2+^	K^+^	Al d	Fe d	Al o	Fe o
			(%)							(mg g^–1^)					
10	BU	${\overline{\text{x}}}$	50.91	36.79	**12.30**	**5.26**	**8.34**	18.76	**6.39**	**6.42**	0.031	**6.66**	**16.35**	12.92	13.60
		SD	11.99	10.45	3.73	0.26	3.47	4.02	1.85	2.74	0.024	3.56	5.13	6.24	4.43
	MO	${\overline{\text{x}}}$	52.70	37.90	**9.50**	**6.08**	**3.64**	17.89	**4.98**	**4.79**	0.025	**4.19**	**11.16**	10.78	11.40
		SD	15.10	14.01	2.84	0.40	2.17	1.94	1.22	1.34	0.010	2.62	5.05	5.26	4.74
20	BU	${\overline{\text{x}}}$	47.45	42.98	**9.57**	**5.58**	**4.81**	17.62	5.71	5.42	**0.016**	**6.55**	**17.21**	**12.96**	**13.15**
		SD	19.23	16.52	4.07	0.25	2.70	3.49	2.12	1.91	0.006	4.30	8.17	6.39	5.46
	MO	${\overline{\text{x}}}$	60.30	33.00	**6.70**	**6.36**	**1.63**	16.61	3.94	4.40	**0.024**	**2.57**	**8.73**	**8.02**	**8.70**
		SD	15.70	14.00	2.20	0.31	0.94	1.52	0.57	0.95	0.009	1.21	3.04	3.14	2.55
30	BU	${\overline{\text{x}}}$	52.00	38.34	9.65	**5.90**	**2.68**	17.14	**4.78**	4.81	**0.016**	**4.81**	**15.30**	**11.57**	**11.35**
		SD	21.95	16.86	6.77	0.22	1.49	2.28	1.59	3.36	0.005	3.36	6.72	6.02	5.15
	MO	${\overline{\text{x}}}$	62.47	31.68	5.86	**6.50**	**1.19**	15.85	**3.74**	4.47	**0.055**	**2.05**	**7.69**	**6.46**	**6.80**
		SD	16.77	15.07	1.89	0.32	0.70	2.58	0.54	0.81	0.118	1.38	2.48	4.26	2.98

Each value was calculated from 15 samples. Values in bold indicate statistically significant differences (p < 0.05) between the two sites for a given property and the same depth.

*BU* Bucharama, *MO* Malyj Orokan, *CL* central location, $\overline{x}$ average value, *SD* standard deviation, *SOCC* soil organic carbon concentration, *HW* hygroscopic water.

## Results

The relatively homogeneous lithological, climatic, and vegetation characteristics of the two research localities gave rise to a practically identical aboveground tree biomass, typical of sparse forest tundra, i.e., 52.2 t ha^−1^ (SD = 37.0 t ha^–1^) for Bucharama and 52.3 t ha^–1^ (SD = 28.5 t ha^–1^) for Malyj Orokan. In contrast, there was a significant difference (p < 0.01) between the average SOL thickness at the former (6.0 cm, SD = 3.3 cm) and the latter localities (15.1 cm, SD = 5.7 cm). A shallow permafrost table was detected in 3 soil profiles in Malyj Orokan at an average depth of 27 cm within the surveyed profile (0–40 cm).

### Soil physical and chemical properties

The soil properties determined in both localities are shown in Table 1. There was a tendency from sandy loam in Bucharma toward loamy sand in Malyj Orokan. The soil pH was slightly acidic in the latter locality and moderately acidic in the former locality. The soil organic carbon concentration at Bucharama was approximately two times higher than that at Malyj Orokan, in agreement with higher concentrations of Al o, Al d, Fe o, and Fe d featuring considerable capacity for SOC binding and stabilization. The soil hygroscopic water content and observed thixotropy were consistent with the SOC content and the presumed presence of allophane and Fe-Al-organic compounds. The majority of average values for variables indicating the advancement of pedogenic processes, i.e., the clay content, soil acidity, and pedogenic Fe and Al, contents decreased with depth. Among exchangeable cations, only the Ca^2+^ content in the top 10 cm was significantly higher in Bucharama, while the amount of highly mobile K^+^ was higher in Malyj Orokan, especially in the 20–30 cm layer. This distribution could be explained by less intense percolation and soil leaching in Malyj Orokan. The C/N ratio (> 13) signaled that in addition to minerally stabilized SOC, particulate organic matter was substantially represented. However, organic matter deposited on the soil surface was unlikely to be affected by cryoturbation, as the average SOC concentration decreased by approximately 50% for every 10 cm depth increment.

### Locality as a random categorical factor

**Figure 2:**
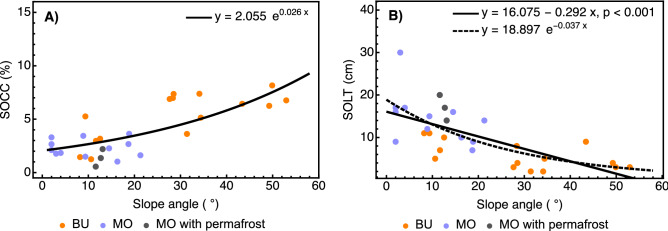
(**A**) The relationship between soil organic carbon concentration (SOCC) and slope angle is fitted by the exponential function. RMSE = 1.528; AIC = 107.523. (**B**) Relationship between surface organic layer thickness (SOLT) and slope angle. Linear fit: adj. R^2^ = 0.445; RMSE = 4.792; AIC = 183.152. Exponential fit: RMSE = 4.584; AIC = 180.487. *RMSE* root mean squared error, *AIC* Akaike information criterion, *BU* Bucharama, *MO* Malyj Orokan.

The separate-slopes ANCOVA tested whether the locality factor comprising geopedological characteristics at Bucharama and Malyj Orokan affected the dependent variable after the influence of the covariates was removed. It showed that when the random effects of the slope angle, altitude, aboveground tree biomass, and SOL thickness were controlled for, the locality as a random categorical factor didn't exert a significant influence on SOC concentration (Table 2). Of all interactions between locality and the covariates, only the interaction with slope angle was significant. The points representing soil profiles in which shallow permafrost tables were observed contributed to the lack of a trend in the interval between 0° and approximately 20°, especially in Malyj Orokan (Fig. 2A). The presence of an unobserved but only slightly deeper permafrost table impacting the soil properties in Malyj Orokan could not be excluded. As a result, the exponential fit appeared to be a more adequate alternative to the linear relationship (Fig. 2A). The slope angle and SOL thickness as covariates with significant or marginally significant interactions with the locality factor were closely related (Fig. 2B): the regression coefficient of SOL thickness on slope angle was significant in Bucharama (b = − 0.126, p = 0.017) and marginally significant in Malyj Orokan (b = − 0.377, p = 0.088), with no significant difference between them. While the relationship across both localities also appeared weakly nonlinear, the small differences between the MSE and AIC values for the linear and exponential fits did not support the superiority of either model.

**Table 2:** Separate-slopes ANCOVA on the effect of locality as a random categorical factor on the cube root-transformed soil organic carbon concentration, adjusted for altitude, slope angle, aboveground tree biomass, and surface organic layer thickness.

Effect	DF	Sum of square	Mean square	F-value	p-value
Intercept	1	0.842	0.842	5.881	0.249
Locality × slope angle	2	0.329	0.164	4.048	**0.033**
Locality × aboveground tree biomass	2	0.017	0.009	0.212	0.811
Locality × surface organic layer thickness	2	0.214	0.107	2.636	0.096
Locality × altitude	2	0.137	0.069	1.691	0.210
Locality	1	0.143	0.143	3.527	0.075

The locality factor represents geopedological characteristics pertaining to soil organic carbon at Bucharama and Malyj Orokan. Values rendered in bold are statistically significant at p < 0.05.

### SOC concentration association with soil biophysical variables

**Figure 3:**
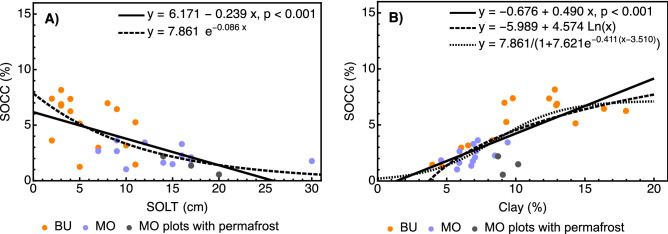
(**A**) Relationship between soil organic carbon concentration (SOCC) and surface organic layer thickness (SOLT). Linear fit: adj. R^2^ = 0.421; RMSE = 1.762; AIC = 123.144. Exponential fit: RMSE = 1.631; AIC = 118.496. (**B**) Relationship between SOCC and the clay content. Linear fit: adj. R^2^ = 0.533; RMSE = 1.581; AIC = 116.636. Logarithmic fit: RMSE = 1.568; AIC = 116.135. Sigmoid fit: RMSE = 1.544; AIC = 117.208. *BU* Bucharama, *MO* Malyj Orokan, *RMSE* root mean squared error, *AIC* Akaike information criterion.

The broader biophysical environment variables included in the MLR analysis were terrain morphology (altitude, slope angle), vegetation and surface organic material (aboveground tree biomass, SOL thickness), and soil structure/texture, represented by the contents of silt, clay, and the content of Ca^2+^ that facilitates the presence of soil aggregates. Multiple stepwise backward linear regression analysis retained SOL thickness (Fig. 3A) and clay content (Fig. 3B) as the best predictors of SOC concentration among all explanatory variables (Table 3). The partial correlation of SOC with Ca^2+^ was relatively high, but the calcium cation did not make a significant contribution to the overall MLR model. Conspicuously, there was no significant correlation between SOC concentration and aboveground tree biomass. The expected increase in SOC concentration with increasing clay content was absent in the profiles featuring shallow permafrost tables (less than 35 cm deep) in Malyj Orokan (Fig. 3B). Because the increase in SOC concentration began to level off at approx. 12% clay content, logarithmic or sigmoid fits were also produced. The linear approximation of the relationship between SOC concentration and the clay content was upheld by relatively low MSE and AIC differences (16% and 4.64, respectively) between linear and nonlinear fitting functions (Fig. 3B), but the nonlinear trend could still reflect the hindrance of some important processes, as discussed further below.

**Table 3:** Stepwise backward selection multiple linear regression of soil organic carbon concentration (SOCC) on biophysical variables: adj. R^2^ = 0.676, p < 0.001.

Dependent variable	Altitude (m)	SA (°C)	ATB (t ha^–1^)	SOLT (cm)	Silt (%)	Clay (%)	Ca^2+^ (mg g^–1^)	Intercept
Equation [SOCC, %]	**–**	**–**	**–**	**− 0.153**	**–**	**0.371**	**–**	1.973
Partial correlation	–0.120	0.163	0.167	**− 0.574**	–0.243	**0.679**	**0.456**	
p value	0.540	0.407	0.395	**0.001**	0.214	** < 0.001**	**0.015**	0.056

*SA* slope angle, *ATB* aboveground tree biomass, *SOLT* surface organic layer thickness.

Numerical values printed in bold are statistically significant (p < 0.05).

### Soil organic carbon concentration association with pedogenic Al and Fe

**Figure 4:**
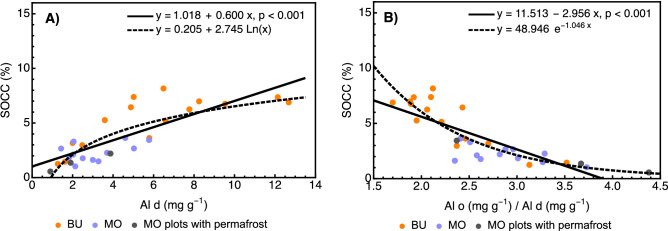
(**A**) Relationship between soil organic carbon concentration (SOCC) and the dithionite-extracted Al d. Linear fit: adj. R^2^ = 0.637; RMSE = 1.396; AIC = 109.160. Logarithmic fit: RMSE = 1.358, AIC = 107.523. (**B**) Relationship between SOCC and the Al activity ratio (oxalate-extracted Al o/dithionite extracted Al d). Linear fit: adj. R^2^ = 0.658; RMSE = 1.355; AIC = 107.361. Logarithmic fit: RMSE = 1.134; AIC = 96.693. *BU* Bucharama, *MO* Malyj Orokan, *RMSE* root mean squared error, *AIC* Akaike information criterion.

The contents of dithionite- and oxalate-extracted Fe and Al were analyzed by multiple stepwise linear ridge regression with backward selection. Only Al d (Fig. 4A) was retained in the final MLR model (Table 4). The predictive value of Al d was supported by its high partial correlation with SOC concentration. Although the RMSE and AIC values did not challenge the formal suitability of the linear fit, the logarithmic fit appeared to reflect some relevant patterns mentioned in the “Discussion”. Additionally, there was a negative SOC concentration regression for the Al o/Al d activity ratio (Fig. 4B), suggesting that the Al-substituted Fe minerals showed a higher affinity for SOC than did Al o, representing mainly poorly crystalline pedogenic Al minerals. Two of the highest Al activity ratios coincided with the presence of a shallow permafrost table, highlighting the role of temperature and liquid water availability in soil weathering. Because of the comparatively low amount of Al d compared to other extractions, the remaining fractions also played an important role in the mineral binding and stabilization of SOC.

**Table 4:** Stepwise backward selection multiple linear ridge regression of soil organic carbon concentration (SOCC) on dithionite- and oxalate-extracted secondary Fe and Al: VIF_max_ = 3.94; adj. R^2^ = 0.575, p < 0.001.

Dependent variable	Al o	Al d	Fe o	Fe d	Intercept
Equation [SOCC, %]	**–**	**0.545**	**–**	–	**1.262**
Partial correlation	0.045	**0.768**	0.350	0.360	
p value	0.819	< 0.001	0.063	0.054	0.013

*Fe d, Fe o, Al o, Al d* dithionite and oxalate extracted Fe and Al, respectively, *VIF*_*max*_ maximum value of the variance inflation factor.

Numerical values printed in bold are statistically significant (p < 0.05).

## Discussion

The lack of soil carbon data complicates the estimation of the total ecosystem carbon storage in subarctic regions affected by global climate change and forest expansion^6^. In particular, the confidence in high-latitude Eurasian soil carbon estimates is only low to medium since the estimates are based on a small dataset^55^. In this context, the present study contributed new information on SOC concentrations and the controls and trends in the highest, NW part of the Central Siberian Tableland built by massive basalt flows. Although geochemical interpretations of continental flood basalts usually assume that individual lava flows are compositionally homogeneous, systematic element variations may occur within a flow field formed by an individual flood basalt eruption^56^. Since the separate-slope ANCOVA did not confirm the effect of locality on SOC concentration when adjusted for biophysical factors, the flow basalts in Bucharama and Malyj Orokan could be regarded as a compositionally homogeneous source of SOC binding and stabilization agents, with mainly active Fe and Al minerals. This result supported our initial assumption about comparable lithologies of the research localities, originally deduced from an example of the homogeneity of lava flows of the Siberian Trap Province^32^. The most obvious explanation for the significant interaction between the locality and slope angle could be derived from the general increase in direct solar radiation with rising slope steepness, typical of high latitudes. For instance, southern slopes at 60°N receive the maximum solar radiation at an approximately 45° inclination, and this maximum amount is nearly 1.5 higher than that on flat surfaces^57^, producing higher temperatures. Increased temperatures in otherwise cold regions lead to more intense vegetation litter decomposition and leaching, improved availability of liquid water, and faster soil weathering^58,59^. The products of litter decomposition, leaching, and soil weathering are crucial for organic matter input into soil and SOC binding and stabilization.

### Soil organic carbon concentration variation

The average SOC concentration on the steep, forest-covered slopes of Bucharama Valley reached 5.2%, slightly more than the values of 4.6% on the mountain tundra plots on the western edges of the plateau^36^ and 4.7% in a low-Arctic site (64°N) featuring relatively flat topography and sparse spruce forest cover but less than the values of 7.6% and 11.7% in sites at considerably lower (58°N) and higher (75°N) latitudes^60^. The average 2.15% SOC concentration on gentler slopes of Malyj Orokan was noticeably lower than that in all the other localities but was comparable to the value of 2.6% in the gently undulating Siberian Yedoma sediments with a SOL thickness of 30–50 cm^61,62^.

Our study results provided a further opportunity to examine the suggestion that SOC concentrations in non-cryoturbated soils of the permafrost region may be similar to those in temperate zone regions^55^. Because SOC concentration can vary by one order of magnitude on a regional scale even under the same type of vegetation or tree species^63^, any likening must account for topography, soil parent material, and vegetation cover. In this context, the average SOC concentration on the slopes of the Putorana Plateau (2.15–5.23%) partly overlapped with a 3–7% interval established in a variety of temperate mountain forest soils derived from claystones, sandstones, mica schist, and ophiolite rocks. However, it was 3–7 times lower than that in soils derived from andesite rock and high in allophanes^63,64^. We deduce that the harsh polar conditions of the Putorana Plateau, i.e., low temperature, limited liquid phase water availability, and low organic matter input did not allow for a full manifestation of the substantial and well-documented capacity of pedogenic Fe and Al minerals for SOC binding and stabilization^15,65^. Instead, it produced SOC concentration values typical of phyllosilicates under much milder conditions in the temperate zone^63^. It appears that interzonal comparisons are relevant not only for the global SOC inventory but also for future SOC-related patterns and trends. The relative compositional homogeneity of the two localities facilitated further analysis of the roles played by some important biophysical factors shaping the observed patterns of SOC concentration, reflecting distinct rates of litter decomposition, soil weathering, and pedogenic processes.

### Soil organic carbon concentration and biophysical factors

The surface organic layer thickness was identified as the most important environmental variable determining the surface SOC distribution (including SOL) in two Siberian tundra and taiga permafrost ecosystems representing relatively flat topography^66^. Additionally, there is a dearth of information on the patterns and the role of the SOL thickness in arctic and subarctic mountain terrains. We found that SOL thickness on the slopes of the Putorana Plateau decreased with increasing slope angle and that clay content and SOL thickness were the best predictors of SOC concentration. Together, the clay content and SOL thickness explained approximately 68% of the total SOC concentration variation. These key associations appeared to be based on several underlying patterns, processes, and relationships, such as the causal link between slope angle and solar irradiation.

In particular, the vegetation litter movement and accumulation in various geographical zones were shown to depend on the topographic position^67,68^. The topographic position, mainly its slope-parallel gravity component defined as the sine value of the slope angle, was an important factor associated with variable SOL thickness across the western Putorana Plateau. The maximum SOL thickness (30 cm) observed at only a 3° inclination in Malyj Orokan corresponded to that in a drained thaw lake basin in Alaska^69^. The average SOL thicknesses in Bucharama (6.0 cm, SD = 3.3 cm) and Malyj Orokan (15.1 cm, SD = 5.7 cm) were similar to the average values reported from two contrasting Siberian localities^66^: 6 cm (SD = 2.8 cm) in a larch taiga situated on a Pleistocene paleoterrace, 480 km south of the Arctic Circle, and 12.3 cm (SD = 8.6 cm) in a tussock polar tundra. In view of the replaceability of ecological factors theory^70,71^, the similarities could have resulted from the partial, temperature-mediated equivalence of slope angle and latitude concerning SOL thickness and its rate of decomposition. A thinner SOL established on steeper slopes most likely accounted for weaker thermal insulation, higher soil temperature, increased percolation, and improved availability of liquid water as important conditions for litter decomposition and soil weathering. Both organic matter leaching^72^ and sufficient mineral weathering^28^ determine the amount of minerally stabilized SOC.

Furthermore, thermal insulation provided by thick lichen/moss and SOL was shown to reduce the thickness of the active layer and the depth of the permafrost table^73^. Long-term measurements from Siberian tundra ecosystems showed that the negative correlation between the surface organic layer thickness and the depth of the active layer is mediated by soil temperature, and its absolute value increases during summer and decreases toward winter^11^. In accordance, we observed shallow permafrost tables (< 35 cm) exclusively on the weakly inclined slopes covered by a comparatively thick SOL at Malyj Orokan (Fig. 2A,B). The average permafrost table depth of 27 cm, observed in several sites under a thick SOL, coincided with the 22–35 cm interval established for a shallow permafrost spot-like occurrence on gentle slopes in the Putorana Plateau's eastern part^30^ under a more continental climate. As mineral soil is largely affected by energy and material flows, the combined influence of its upper and lower boundaries, formed by the SOL and permafrost, has a great potential to affect soil weathering and SOC sequestration by thermal insulation and upward cooling^74^. In this case, the shallow permafrost table is a responder to and a driver or inhibitor of ecosystem characteristics and processes simultaneously. For example, the inhibiting effect of the shallow permafrost, protected by a thick SOL, was likely mediated by a prolonged soil freeze, lasting far into the summer period and hindering soil weathering and the percolation of the litter leachate, containing organic acids and dissolved organic carbon from the SOL, through the mineral soil layers. However, the apparent alignment of both the logarithmic and the sigmoid fits with the data (Figs. 3A,B, 4A) could imply that soil weathering and the supply of the organic matter from the SOL were decoupled with respect to SOC even under more favorable conditions at Bucharama. One explanation could be that the dissolved organic matter adsorption on secondary minerals was hampered by the fast leaching of plant litter degradation products during freshet and its very short residence time, reported from the shallow soils of northern Eurasia that are active only during 2 to 4 summer months^72^. As a result, the possible sequestration of additional organic matter supply resulting from the climate change-driven expansion of the upper forest boundary observed in the Putoran Plateau^6^ could be severely limited.

The content of Ca^2+^ was a weaker predictor of SOC concentration than was the clay content and SOL thickness, and it was not included in the final MLR model. Normally, the role of Ca in SOC binding is reduced as pH shifts from basic to acidic conditions^75^, established in both localities. The consistency of the presented associations among slope angle, SOL thickness, and SOC concentration strongly supports their interpretation as causal interrelationships. Therefore, we concluded that the combined effect of the main biophysical factors, including the slope angle, SOL thickness, and permafrost table position, on SOC concentration was mediated by temperature and the availability of liquid water.

### Organic carbon association with the mineral fraction

Pedogenic Fe and Al play a paramount role in SOC binding and stabilization based on the diverse relative affinities of pedogenic Fe and Al for organic matter^76^. In our study, Al d was the best predictor of SOC concentration among dithionite and oxalate Fe and Al extractions. This instance adds to several other cases where Al showed a higher affinity for SOC than did Fe^77–79^ or it was, as Al d, associated with more favorable weathering conditions^80,81^. Given these findings, we deduced that the higher Al d content in Bucharama resulted from faster soil weathering on steeper slopes and under a thinner SOL. Although we were unable to pinpoint the specific origin of Al d, the Al-substituted Fe oxides were considered its main source^82,83^. The distinctive characteristics of dithionite-extracted Al were observed despite its comparatively lower content, and given the higher proportions of Fe d, Fe o, and Al o, these extractions were responsible for binding the bulk of the minerally stabilized SOC.

The capacity of soils to store C changes during their development since soil weathering initially produces metastable, reactive non-crystalline minerals capable of adsorbing high amounts of SOC that later transform to stable, less reactive crystalline products^84^. The SOC concentration in several basaltic soils of similar age as in the Putorana Plateau, also covered by natural forests, reached approx. 17% in Hawaiian soil (16 °C MAT, 2500 mm MAP)^84^ and 4.7% (8.3 °C MAT, 1150 mm MAP) to 2.4% in southern Cascade Range soils (6.5 °C MAT, 1340 mm MAP)^26^. The last two respective values were quite similar to those in Bucharama (5.27%) and Malyj Orokan (2.13%), where the temperature- and moisture-mediated biophysical controls of slope angle and SOL thickness on litter decomposition, soil temperature, and liquid water availability (due to lesser SOL interception and shorter soil freeze) acted in lieu of climate factors. While the ratio of the Fe + Al oxalate vs. dithionite extractions was higher in Malyj Orokan (1.43) than in Bucharama (1.13), the latter locality featured a higher SOC concentration, associated with faster litter decomposition and a larger input of dissolved organic carbon into soils on steeper slopes, with an approximately 1.4 times higher absolute amount of oxalate-extracted poorly crystalline minerals, and, importantly, with the twofold amount of Al d as the best predictor of SOC concentration.

In addition to the SOC variation explained by mineral binding and stabilization, the remaining variability could be accounted for by the particulate organic matter whose presence was implied by the C/N ratio > 13 (15.85–18.76). In Siberian permafrost soils, particulate organic matter may represent approximately 19% of the bulk organic carbon in mineral horizons^28^. Given the coarse textural composition of Putorana soil, the maximum content of hygroscopic soil water was quite high and consistent with the presence of amorphous allophanes and Fe-Al-organic compounds detected in river water samples from adjacent regions^25^. In a broader sense, our research has confirmed that both oxalate- and dithionite-extractable Fe and Al values are useful in studies of soil formation in subarctic regions^85^.

## Conclusions

In relation to previous studies from the Southern Hemisphere^80,86^, our research produced new findings from the subarctic zone and cast fresh light on the importance of Al d as a SOC concentration predictor and soil weathering indicator in basalt-derived soils of cold regions. The presented investigation addressed the existing information gap regarding interrelationships among relief, vegetation, and SOC concentration in polar mountains. Insufficient soil organic carbon data complicate efforts to detect temporal changes and to derive projections that are potentially deducible by analogy with processes in rapidly warming Arctic and subarctic regions. Our results provide new SOC concentration data from the northern boundary of the forest biome sheltered by the rugged Putorana Plateau. Equally important, the findings revealed several important relationships among slope angle, SOL thickness, free pedogenic Fe and Al, and SOC concentration. In particular, slope angle and SOL thickness were shown to have paramount effects on the soil weathering rate and SOC concentration, and these effects were mediated by solar irradiation, thermal insulation, and the interception of water percolating toward mineral soil. The acquired regression equations explaining a significant part of SOC concentration variability could produce SOC estimates for the vast basaltic territory of the Central Siberian Tableland and other basalt flows in polar areas using slope angle, surface organic layer thickness, and clay content as the input values derived from the geomorphological causality in the distribution of soil and vegetation types. These and other biophysical factors are for instance obtainable from the remote sensing-based multipurpose large-scale mapping that was described and tested earlier in another part of the Putorana Plateau^30^.

## Data Availability

The datasets analyzed during the current study are available from the corresponding author upon reasonable request.
